# Mechanistic Implications of Biomass-Derived Particulate Matter for Immunity and Immune Disorders

**DOI:** 10.3390/toxics9020018

**Published:** 2021-01-20

**Authors:** Arulkumar Nagappan, Su Bum Park, Su-Jun Lee, Yuseok Moon

**Affiliations:** 1 Laboratory of Mucosal Exposome and Biomodulation, Department of Integrative Biomedical Sciences, Pusan National University, Yangsan 50612, Korea; arulbiotechtnau@gmail.com; 2 Department of Internal Medicine, College of Medicine and Research Institute for Convergence of Biomedical Science and Technology, Pusan National University Yangsan Hospital, Yangsan 50612, Korea; psubumi@hanmail.net; 3 Department of Pharmacology, Inje University College of Medicine, Busan 47392, Korea; 2sujun@inje.ac.kr; 4 Biomedical Research Institute, Pusan National University, Yangsan 50612, Korea

**Keywords:** particulate matter, biomass, immunity, host defense, hypersensitivity

## Abstract

Particulate matter (PM) is a major and the most harmful component of urban air pollution, which may adversely affect human health. PM exposure has been associated with several human diseases, notably respiratory and cardiovascular diseases. In particular, recent evidence suggests that exposure to biomass-derived PM associates with airway inflammation and can aggravate asthma and other allergic diseases. Defective or excess responsiveness in the immune system regulates distinct pathologies, such as infections, hypersensitivity, and malignancies. Therefore, PM-induced modulation of the immune system is crucial for understanding how it causes these diseases and highlighting key molecular mechanisms that can mitigate the underlying pathologies. Emerging evidence has revealed that immune responses to biomass-derived PM exposure are closely associated with the risk of diverse hypersensitivity disorders, including asthma, allergic rhinitis, atopic dermatitis, and allergen sensitization. Moreover, immunological alteration by PM accounts for increased susceptibility to infectious diseases, such as tuberculosis and coronavirus disease-2019 (COVID-19). Evidence-based understanding of the immunological effects of PM and the molecular machinery would provide novel insights into clinical interventions or prevention against acute and chronic environmental disorders induced by biomass-derived PM.

## 1. Introduction

Particulate matter (PM) is a term used to describe a mixture of pollutants, containing a complex mixture of smoke, dust, and other solid particles, as well as liquid droplets, present in the air [1]. PM vary in size, shape, and chemical composition. PM is not just carbon black, but is also a carrier of some detrimental factors, including heavy metals, allergens, microbial components, and other organic toxicants [2,3]. The World Health Organization (WHO) and the International Agency for Research on Cancer (IARC) have shown that airborne PM can contain Group 1 carcinogens [4,5,6]. Following the inhalation of PM into the human airway, the airway epithelial layer is the first barrier to systemic exposure. In addition to airway distress, including dyspnea, cough, and aggravation of asthma, exposure to PM has been closely associated with the risk of lung cancer [7,8]. Additionally, PM is responsible for chronic respiratory diseases, such as chronic obstructive pulmonary disease (COPD) [9,10,11]. PM can penetrate different tissues and cause severe oxidative damage wherever deposited [12,13]. Furthermore, circulatory exposure to PM through the respiratory tract is directly linked to an elevated risk of arrhythmia, myocardial infarction, premature delivery, low birth weight, and even premature death in cardiovascular or respiratory comorbidities [14,15,16,17]. In particular, the immune system is a sensitive tissue to airway PM and allergen exposure and is involved in diverse tissue disorders. Based on the hypothesis that immune responses are closely linked to airway and systemic distress during PM exposure.

It has been well known that biomass fuel combustion, as well as wildland and agricultural fires, exhale large quantities of harmful air pollutants to both ambient and indoor air, which is associated with increased incidences of respiratory disease-related morbidity and mortality [18,19]. Exposure to PM derived from woodsmoke causes various health effects, such as systemic and airway inflammation, and increases the incidence of asthma and allergic diseases [20,21,22]. Furthermore, exposure to wood smoke particles (WSP) is associated with various types of lung distress, including acute respiratory infection (ARI) and COPD [23,24,25,26].

Many previous studies addressed the health effects of different types of PM exposure [27,28,29,30,31]. Although investigations attempted to assess the effect of PM on the immune systems [32,33], mechanistic pathways involved in immune alteration-related pathologic events have not been fully established. In this review, we systemically discussed the immune alteration-related events and associated the disease outcomes with cellular and molecular evidence in humans and animal models in response to PM, including biomass-derived pollutants. The comprehensive review would provide insights into strategies of prevention and novel intervention with environmental immune diseases.

## 2. Source and Characteristics of PM

PM is a mixture of solid and liquid particles suspended in the air, varying in size, number, surface area, chemical composition, and sources. Among the various methods of PM classification, the aerodynamic diameter is one appropriate criterion to define transportability in the atmosphere and inhaling ability through the respiratory tract. PM is categorized as PM_10_, PM_2.5_, and PM_0.1_, according to mean particulate size fractions. Based on “aerodynamic equivalent diameter” (AED), PM is categorized into three classes, including coarse particles or PM_10_ (ranging from 2.5 to 10 μm); fine particles or PM_2.5_ (smaller than 2.5 μm); ultrafine particles or PM_0.1_ (UFP, smaller than 0.1 μm) [34,35]. Regardless of the PM composition, differently sized PMs are expected to have different particle velocities for settling in the respiratory tract [36]. Generally, PM contains various chemical constituents, including inorganic ions (e.g., sulfates, nitrates, ammonium, sodium, potassium, calcium, magnesium, and chloride), heavy metals (including cadmium, copper, nickel, vanadium, and zinc), black carbon, and organic compounds (e.g., polycyclic aromatic hydrocarbons, and polyaromatic hydrocarbons (PAHs)) [28,37,38]. The main sources of coarse particles (PM_10_) include abraded soil, dust from road and construction sites, and oil combustion products with bio-aerosols, such as fungi, bacteria, pollen, and endotoxins. Particles that are derived from direct emissions owing to combustion processes of gasoline, oil, diesel, wood burning, and coal burning are primary sources of fine (PM_2.5_) and ultrafine particles (PM_0.1_) [38,39]. The woodsmoke poses the pollutants like carbon monoxide (CO), nitrogen oxides (NOx), methane, chlorinated dioxin, volatile organic compounds (VOC), PAH, and PM [18,40,41]. The general properties and primary sources of fine (PM_2.5_) and coarse (PM_10_) particles are described in Table 1.

### Particle Size-Dependent Translocation and Toxicity in the Airway Mucosa

PM_2.5_ contains high levels of transition metals associated with increased potential of oxidative stress in the tissue. Moreover, PAHs, richly distributed in UFP (PM_0.1_), can generate oxidative stress, whereas their levels are low in both coarse (PM_10_) and fine (PM_2.5_) particles [33]. Generally, PM_10_ settles in the upper respiratory tract and is substantially associated with several diseases, including cardiovascular diseases [42,43], cancers [7,44,45], tuberculosis [46,47], and asthma [48,49]. Furthermore, PM_2.5_ induces vascular inflammation, leading to a potentially increased risk of atherosclerotic cardiovascular complications [50]. In addition to airway disorders, another susceptible target of circulating PM is the cardiovascular system as the incidence of ischemic heart disease and cardiac injury increases with PM exposure. A study investigating fine particles and cardiac injury revealed that PM_2.5_ exposure led to a substantial increase in interleukin 4 (IL-4) and IL-13 levels and a decrease in interferon γ (IFN-γ) in the myocardium of rats [51]. Taken together, the particulate size is the deterministic factor of translocation and actions in the airway mucosa and other tissues.

## 3. Mucosal Exposure and Innate Immune Responses to PM

### Innate Immune Regulation and Signaling in the Lung Barrier

**Figure 1:**
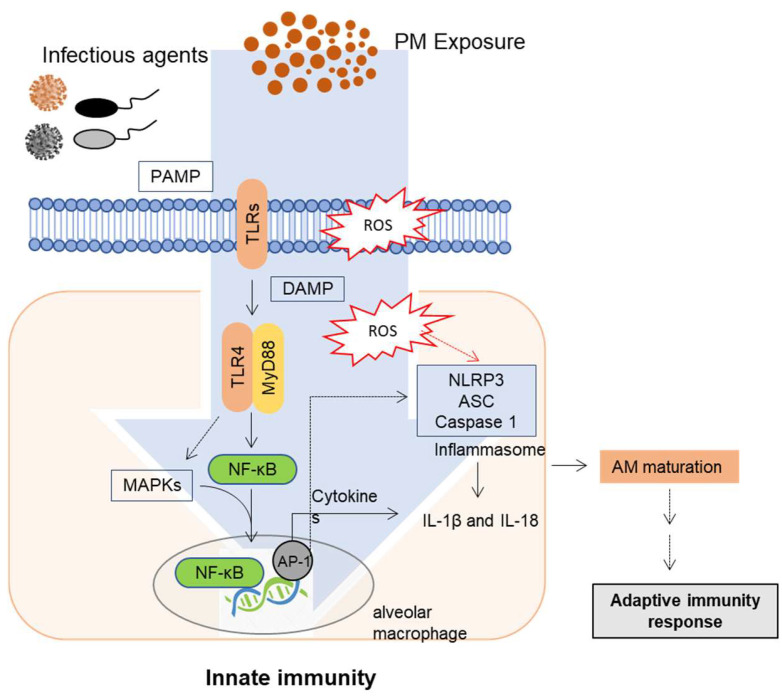
A schematic diagram of PM exposure-induced innate immune responses. Lung mucosal exposure to PM can affect the innate immunity-associated human disease outcomes. PM exposure alters pattern recognition receptor (PRR)-linked innate immunity and subsequent adaptive immunity to infectious agents or allergens. During the pathologic process, various types of endogenous molecules, including reactive oxygen species (ROS) and cytokines, are involved in pulmonary tissue injuries or systemic immune dysfunctions via circulation. In particular, PM-insulted disruption of immunity may lead to infection, hypersensitivity, and chronic disorders. Excess oxidative stress can change the profile of Th cell-polarizing cytokines. Moreover, PM exposure can alter the expression of key transcription factors (GATA3 and T-bet) and disrupt the balance between Th1 and Th2 cells, influencing the disease severity.

When biomass burning-derived ultrafine and fine particles enter into the airway through inhalation from ambient air, they can reach the alveolar tissue. Compared to most deposited particles, ultrafine particles can cross the airway-blood barrier and enter the pulmonary and systemic circulation [52,53,54]. Although most inhaled PM_0.2_ and PM_2.0_ particles are deposited in the acinar region of the lung, some of them can be released into circulation with time and can be found in the kidney and liver [55]. The well-known mechanism of nanoparticles passing through the air-blood barrier in the acinar region is the process of endocytosis or diffusion [56,57,58]. However, the delivery of PM_0.2_ and PM_2.0_ particles to blood circulation and extrapulmonary organs might be mediated by particle-internalizing macrophages [55]. Following the inhalation of pollutants, such as PM into the human airway, airway epithelial cells are the first defense of the respiratory system. When barrier epithelial cells are exposed to PM (especially combustion-derived PMs), oxidative stress and inflammation are elevated in the lung [59], thus disrupting epithelial barrier integrity. Moreover, smooth muscles are thickened, and the epithelial-mesenchymal transition is induced [60]. Through the epithelial-mesenchymal transition, lung epithelial cells display reduced levels of E-cadherin, representing the loss of characteristic features of epithelial polarity and intercellular junctions. In addition to barrier disruption, the innate immune responses to PM in human airway epithelial cells and alveolar macrophages are affected by changes in pattern recognition receptors (PRRs), including Toll-like receptors (TLRs) and nucleotide-binding domain and leucine-rich repeat (NLR) containing inflammasomes [61,62,63,64]. Household air pollution from biomass fuel combustion triggers dose-dependent production of proinflammatory cytokines and alters phagocytosis activity in human alveolar macrophages [65]. TLRs are representative PRRs that play pivotal roles in innate immune responses to infection in epithelial cells and phagocytes. The mechanisms involved in PM-induced innate immune responses are mainly through the production of proinflammatory cytokines and chemokines via activation of the TLR signaling pathway [1,66,67]. There is ample evidence demonstrating that PM modulates cytokine and chemokine levels, although the effects vary depending on the airway exposure regime [61,68]. TLRs can modulate myeloid differentiation primary response protein 88 (MyD88), resulting in the activation of the nuclear factor of kappa light polypeptide gene enhancer in B-cells inhibitor, alpha (IκBα) and nuclear factor kappa B (NF-κB) inhibitory protein, which activates the expression of proinflammatory genes [69,70,71,72,73]. Moreover, airway epithelial cells exposed to PM_10_ can activate the NLRP3 inflammasome, subsequently facilitating the release of a mature form of IL-1β and IL-18, whose intracellular expression was induced by NF-κB signaling [64]. Furthermore, PM_2.5_ can activate TLR4 and the NLRP3 inflammasome in cooperation with mitogen-activated protein kinase (MAPK) pathways in monocytes [61]. IL-1β can recruit immune cells, including neutrophils and lymphocytes [74,75]. Additionally, PM induced granulocyte/macrophage colony-stimulating factor (GM-CSF) and CC chemokine ligand (CCL)-20 production. Notably, GM-CSF can recruit mature dendritic cells (DCs) [76,77,78]. The expression of GM-CSF, leukemia inhibitory factor (LIF), IL-1α, and IL-8 was reportedly increased via PRR action induced by PM_10_ exposure [78,79]. Therefore, ambient PM-activated PRR signaling has a pivotal role in early proinflammatory cell activation and recruitment for further immune responses in the human lung, as well as in circulation. Further studies are required to clarify specific components of the PM mixture that activate PRR signaling during early innate immunity and proinflammatory responses (Figure 1).

## 4. Adaptive Immune Response to PM Exposure

### 4.1. Influence of PM Exposure on T Cell Population

Herein, we focused on the relationship between adaptive immunity and exposure to PM by reviewing recent studies with mutual interests. Currently, most studies have revealed that T lymphocyte-mediated adaptive immune responses are associated with the pathophysiology following PM exposure. CD4^+^T cells, also known as T helper cells (Th cells), are key components of the adaptive immune system. The Th cells can be divided into three types, such as Th type-1 (Th1), Th type-2 (Th2), and Th type-17 (Th17) cells, displaying unique cytokine secretion phenotypes with distinct functional characteristics [80]. After the differentiation of Th cells, Th1 and Th2 cells play distinct roles in immune responses. Th1 cells are triggered by the polarizing cytokine IL-12 and secrete IFN-γ and IL-2, facilitating the cell-mediated response, typically against intracellular bacteria and protozoa [81]. Th2 cells are triggered by the polarizing cytokines IL-4 and IL-2, and are involved in humoral immune responses via their effector cytokines, such as IL-4, IL-5, IL-9, IL-10, IL-13, and IL-25 [80,82]. Several studies have investigated the influence of PM on Th1/Th2 responses, suggesting diverse answers to this question. First, some studies revealed that the Th1 profile can be affected by PM [28,83,84]. Levels of IL-12 and IFN-γ, the Th1 cytokines, were increased in a dose-dependent manner following PM exposure [84,85]. Additionally, the production of numerous cytokines, including IL-6, IL-1β, and GM-CSF, except IL-10, increased in human alveolar macrophages (AMs) exposed to PM_10_ [67,86,87], which accounts for alterations in the T cell cytokine-mediated cellular immunity in response to PM in the airway mucosa.

PM can also affect Th2 cytokine profiles. For example, diesel extract particles (DEP) favor Th2-type responses, whereas black carbon particles upregulate both Th1 and Th2 cytokine production [88]. Moreover, fine particles induced lung inflammation through the production of IL-13 and IL-25 in alveolar macrophages [89]. IL-13 is structurally and functionally similar to IL-4 and plays an important role in the defense against parasite infections and allergic diseases. Furthermore, IL-13 promotes fibrosis in the chronic inflammation state and stimulates mucus production by pulmonary epithelial cells [90,91,92]. In addition, IL-13 affects B cells to induce isotype switching, resulting in a switch from B cells to immunoglobulin E (IgE) [93]. IL-25 is structurally similar to IL-17 and is produced by several cell types, including T cells, mast cells, and macrophages. IL-25 induces the production of other Th2-type cytokines, such as IL-4, IL-5, and IL-13 [94,95]. When PM and specific allergens are inhaled simultaneously, T cell-mediated inflammation increases IL-4, IL-5, IL-13, GM-CSF, chemokine (C-C motif) ligand 5 (CCL5), monocyte-chemotactic protein-3 (MCP-3), and macrophage inflammatory protein-1 (MIP-1) [88]. Moreover, the upregulation of transcription factor GATA Binding Protein 3 (GATA3; Th2 maker) and the downregulation of T-bet (Th2 maker) have been identified in the nasal mucosa of PM_2.5_-exposed rats [96]. An increase in Th2-related cytokines and a decrease in Th1-related cytokines disrupt the balance between Th1 and Th2 cells, with skewing toward Th2, which is closely related to immunotoxicity in the cardiovascular system. Therefore, depending on the exposure regimes and host factors, differential regulation of helper T cell population is markedly involved in PM-induced immune regulation.

#### 4.1.1. AMs as Pivotal Mediators of T Cell Skewing

As stated above, PM_2.5_ disrupts the balance of Th1/Th2 cells. Numerous studies have revealed that this imbalance is induced at the transcriptional level, as well as by various cytokines [97,98]. AMs are known as the principal cells that process airborne particles in the lung and act as APCs to activate the adaptive immune response, although not as efficiently as DCs [99]. Hence, we postulate that antigen presentation stimulates immature T lymphocytes in the respiratory tract to become either mature helper T cells or mature cytotoxic T cells. Moreover, major histocompatibility complex (MHC) II expression by macrophages is an important part of presenting antigens to stimulate T cells. Expression level of MHC II increases when AMs phagocytize PM containing endotoxins, such as lipopolysaccharide [100]. Reportedly, PM promotes B7 and cluster of differentiation 40 (CD40) expression in DCs and monocytes [101,102,103]. Therefore, naïve T cells can differentiate into Th1 or Th2 cells. Some studies have reported that PM induces an imbalance of Th1/Th2 via TLR-MyD88 signaling [62,63]. Additionally, studies have reported that the TLR4-MyD88 dependent pathway plays an important role in recognizing the PM_2.5_ and Th2 differentiation process [62,63]. PM affects transcription factors, such as NF-κB, which play a pivotal role in adaptive immune responses. Activation of NF-κB promotes T cell activation, proliferation, and survival [104]. Several studies have reported that the activation of NF-κB correlates with the size and composition of PM [105,106,107]. On fulfilling specific conditions, NF-κB signaling can be upregulated, and MHC II and costimulatory molecules can be sequentially activated.

#### 4.1.2. Adjuvant-Like Actions of PM in T Cell Regulation

Reportedly, the effect of PM as an adjuvant has been assessed on the adaptive response [108]. In this context, an adjuvant is a substance that boosts the immune response to antigens on treatment with antigens. Therefore, PM_2.5_ was administered only during the sensitization period with house dust mite (HDM) as the allergen. The results showed that PM acted as an adjuvant by promoting inflammation and allergic sensitization, whereas other groups failed to induce important inflammatory responses. Another notable result was that the PAH content in PM promoted Th17-responses and led to the activation of the aryl hydrocarbon receptor (AhR). PM exposure stimulated the expression of Th17 cytokines, including IL-17 and IL-22, as well as that of retinoic acid-related orphan receptor gamma t (RORγt), the Th17 lineage determining transcription factor. Following allergen sensitization, the stimulated expression of AhR in DCs promoted T lymphocyte activation and differentiation into Th17 cells. Furthermore, PM induced allergic sensitization via Th2 associated inflammation [108]. Th17 subsets are involved in the recruitment of neutrophils and induction of inflammation in response to PM exposure.

### 4.2. PM-Induced Suppression of T Cell Immune Responses

Some reports have demonstrated that PM exposure suppressed T cell-mediated cellular immune responses [59,60,78], which poses a risk of respiratory infection. DC is the APC capable of initiating the primary T cell response. In the lung, local DCs react with the PM that penetrates the respiratory tract barrier, bridging innate immunity with adaptive immunity by antigen presentation. Although PM enhanced GM-CSF-induced DC maturation, it attenuated IL-5, IL-13, and IFN-γ secretion, thus lowering the frequencies of alloantigen-specific T helper type 1 effector cells [78]. This indicates that PM exposure may impair the T helper 1 response and increase susceptibility to pulmonary pathogens [78]. In response to PM exposure, DCs tend to mature into distinct cell phenotypes, which promote immune suppression rather than immune stimulation [109]. These tolerogenic, immunosuppressive DCs significantly decreased T helper type 2 and significantly increased regulatory T cells.

Furthermore, accumulated evidence has revealed that PM can deteriorate the host immune system by activating regulatory T cells and IL-10. In vivo experiments in neonatal mice showed that environmentally persistent free radicals significantly increased the levels of pulmonary regulatory T cells and immunosuppressive cytokine IL-10 following influenza infection [110,111]. Consequently, the protective T cell function was suppressed, and the disease severity of influenza was enhanced, indicating that PM exposure may interfere with human immune suppression via regulatory T cells and immunosuppressive cytokines [110,111]. In mice, compared with filtered air (FA) exposed/HDM-challenged mice, UFP-exposed offspring demonstrated lower white blood cell counts in the bronchoalveolar lavage fluid, as well as less pronounced peribronchiolar inflammation. Furthermore, mice prenatally exposed to UFP significantly elevates circulating IL-10 levels, resulting in the upregulation of regulatory T cells and reduced levels of inflammatory cytokines, IL-13 and IL-17, suggesting suppression of Th2/Th17 responses [112]. Collectively, these findings verify the lowered immune response against allergens and pathogens in PM-exposed subjects.

### 4.3. Controversy Regarding the Effect of PM on Adaptive Immunity

PM has been well known to affect public health and is considered an economic burden, especially in developing countries. Several scientific studies have confirmed that the pathophysiology of this process is associated with adaptive immunity [33,62,63,96]. Previous studies have suggested that PM stimulates adaptive immunity; conversely, some findings report that PM inhibits the adaptive immune response [109,110,111]. These two opposing results may seem problematic, but the research we introduced is not mutually exclusive. As experiments are performed in distinct settings in different laboratories, PM can either stimulate or suppress immune responses by T lymphocytes. Further studies are necessary to determine factors that determine the balance between stimulation and suppression in PM-associated adaptive immunity. The composition or size of the PM, age of the patient or laboratory animals, and degree of exposure are possible factors that should be closely monitored.

## 5. Effects of PM on Host Resistance to Infection

### 5.1. Roles of Airway Macrophages in Response to Infection and PM Exposure

For microbes to establish infection in the lower respiratory tract, they must first avoid the innate immune system, composed of the epithelial cell barrier, antimicrobial molecules in mucus, AMs, neutrophils, natural killer cells, and DCs. AMs detect pathogen-associated molecular patterns via TLRs. Through TLR activation, a pathogen-triggered immune reaction cascade is stimulated [32,33]. This cascade includes cytokine release, AMs, and reactive oxygen species (ROS) originating from neutrophils, phagocytosis, and intracellular killing. An excessive immune response could destroy barrier defense and increase the risk of developing other infections, such as tuberculosis [113].

**Figure 2:**
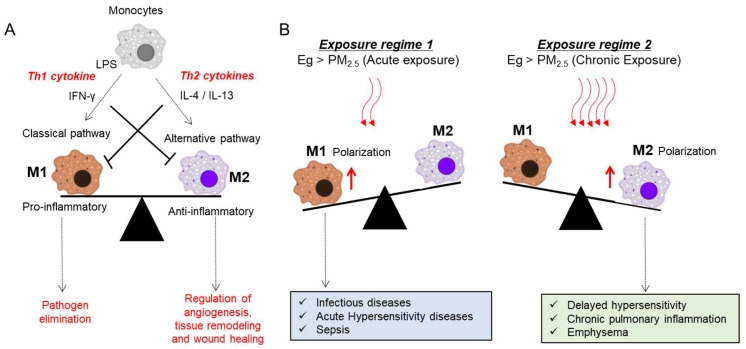
Impacts of PM on macrophage polarization and disease outcomes. (**A**) There are two kinds of macrophages. Classically activated macrophages (M1) and alternatively activated macrophages (M2) are functionality corresponding to the Th1/Th2 paradigm. Macrophages differentiate into M1 type in response to INF-γ from Th1 cells and LPS, and M2 type in response to IL-4 and IL-13 from Th2 cells. Both M1 and M2 macrophages have a distinct phenotype, and they release pro-and anti-inflammatory cytokines, respectively. (**B**) Exposure to PM induced polarization towards M1 (exposure regime 1) or M2 (exposure regime 2) is associated with various respiratory and systemic immune disorders.

Macrophages act as the first line of defense in recognizing tissue injury in response to PM or microorganisms. Macrophages are mainly involved in the process of phagocytosis, and eradication of bacteria, as well as other harmful organisms [11]. In general, monocytes can be differentiated into either activated macrophage M1 or M2 with distinct phenotypes and functionality corresponding to the Th1/Th2 paradigm [114]. Many Th1-derived proinflammatory cytokines are involved in activating M1 macrophages, which can mediate host defense against the various infections of bacteria, viruses, and protozoa. The main function of M2 macrophages is an anti-inflammatory response by stimulating adaptive Th2 immunity, as well as the regulation of angiogenesis, tissue remodeling, and wound healing [114]. An imbalance of M1/M2 polarization is associated with the development of various immunological disorders [115,116]. Therefore, it is pivotal to maintain the balance between M1 and M2 macrophages for the host homeostasis. Exposure to PM_2.5_ induces M1 macrophage-dependent inflammation by increasing secretions of IFN-γ, and IL-17 and IL-21 in CD4^+^T cells, indicating activation or enrichment of Th1 and Th17 responses, respectively [117]. Mechanistically, exposure to PM_2.5_ particles promotes M1 polarization through TLR4-activated JNK and p38 pathways [118]. In contrast, chronic exposure to PM_2.5_ reprograms cellular polarization toward M2 macrophage-involved adverse outcomes, including sustained inflammation and emphysematous lesions. Moreover, chronic exposure to PM_2.5_ downregulates the expression of histone deacetylase 2, which is crucial for deleterious airway remodeling, facilitating COPD [119]. Depending on the exposure regimes, differentially activated macrophages play crucial roles in disease development and progression via specific molecular pathways (Figure 2).

### 5.2. PM Exposure in Bacterial Infection

During bacterial infection, it is recognized by their PRRs like TLRs activates macrophages to produce M1-like proinflammatory cytokines, such as TNF-α, IL-1, IL-6, IL-12, and NO that can eliminate the invading bacterial pathogens [120]. Some bacterial pathogens prevent M1-like polarization, in that case driving the polarization toward an M2 phenotype to lessen the inflammatory response. *Mycobacterium (M.) tuberculosis* infection induces M1 polarization, which secretes the proinflammatory mediators, such as TNF-α, IL-6, IL-12, CCL5, and chemokine C-C motif ligand (CXCL18) [121]. However, during the formation and development of tuberculous granulomas, the active state of macrophages underwent M1-like to M2-like transition [122].

A potential link between long-term exposure to suspended PM and increased rates of pulmonary tuberculosis (TB) has been reported [47,123,124]. To address the effects of PM on the natural course of tuberculosis infection, researchers reported that PM impaired human peripheral blood mononuclear cell (PBMC) immune reactions toward *M. tuberculosis*, via the downregulation of TLR-dependent cytokines [47]. More specifically, DEP as a part of urban ambient PM_2.5_ interfered with human anti-mycobacterial immunity [28,46]. Following the exposure of PBMCs to DEP, researchers observed the suppression of expression of *M. tuberculosis*-induced TLRs 3, 4, 7, and 10, as well as NF-κB-induced expression of chemokines and cytokines, including IFN-γ, TNF-α, IL-1β, and IL-6, which play an important role in the control of *M. tuberculosis*. In vitro and in vivo *M. tuberculosis* infections promoted epithelial cells in the airway, provoking anti-mycobacterial reactions, including the release of cytokines, chemokines, and antimicrobial peptides. A549 cells, a type II alveolar epithelial cell line, generated IL-8 and MCP-1, which attract neutrophils, T cells, basophils, and monocytes, in the early stages of *M. tuberculosis* infection [46]. Furthermore, A549 cells produce human β-defensin 2 (BD-2) and 3 under the influence of mycobacterial lipids and different *M. tuberculosis* strains. Human BD-2 and 3 are tiny cationic peptides that are affiliated with a family of antimicrobial peptides. As they possess antimicrobial characteristics and modulate the immune response, human BD-2 and 3 play key roles in the early control of *M. tuberculosis* infection by reducing the pulmonary bacterial load [46].

On investigating PM_2.5_ and T cell functions in human PBMCs, it has been observed that urban PM may negatively affect the mechanisms of *M. tuberculosis*-specific human T cell functions [47,125]. Exposure to PM_2.5_ reduced the capacity of PBMCs to restrict the growth of *M. tuberculosis* and downregulated the expression of the early activation marker, CD69, during infection on CD3^+^T cells. Furthermore, these findings suggest that PM_2.5_ exposure decreased the production of IFN-γ in CD3^+^, TNF-α in CD3^+^, and CD14^+^ PBMC, which were infected by *M. tuberculosis*, as well as the *M. tuberculosis*-induced expression of T-box transcription factor (TBX21; T-bet) [47,125]. Notably, in contrast to these results, PM_2.5_ exposure increased the expression levels of anti-inflammatory cytokines, such as IL-10 in CD3^+^ and CD14^+^ PBMCs. These data revealed that PM_2.5_ damaged crucial anti-mycobacterial T cell immune functions [125]. Another study investigated whether in vitro exposure to urban air pollution PM alters human immune responses to *M. tuberculosis* in human PBMCs obtained from IFN-γ release assay (IGRA+) and IGRA− healthy study subjects. This study was performed in Iztapalapa, a highly populated tuberculosis-endemic municipality of Mexico City with elevated outdoor air pollution levels, and researchers collected monthly PM_2.5_ and PM_10_ from rainy, cold-dry, and warm-dry seasons to evaluate whether seasonal changes affect the human immune response to *M. tuberculosis*. This result corroborated previous research findings, demonstrating that, except for PM_2.5_ from the cold-dry season, pre-exposure to all seasonal PM reduced *M. tuberculosis* phagocytosis by PBMC. Additionally, *M. tuberculosis*-induced IFN-γ production was suppressed in PM_2.5_ and PM_10_ pre-exposed PBMC from IGRA+ subjects. Hence, both pre-exposure to PM_10_ and PM_2.5_ resulted in a greater loss of *M. tuberculosis* growth control [47] As there exists a close mechanistic association between PM exposure and response to *M. tuberculosis*, the epidemiological evidence in populations with high tuberculosis prevalence should be explored, including the Korean peninsula, who also present a high risk of PM exposure.

### 5.3. PM Exposure in Viral Infection

A previous study has demonstrated that high doses of PM_10_ suppressed respiratory tract inflammatory responses to the respiratory syncytial virus (RSV), a frequent cause of viral pneumonia in infants and the elderly [126]. Therefore, the hosts displayed lower viral immunity after simultaneous exposure to RSV and PM, compared with the defense against a single infection by RSV. Coronavirus disease 2019 (COVID-19), a novel disease, has emerged as a major public health concern. The primary symptoms of COVID-19 include respiratory tract symptoms, such as fever, cough, fatigue, pneumonia, and acute respiratory distress syndrome, remaining asymptomatic in some cases [127]. Severe acute respiratory syndrome coronavirus 2 (SARS-CoV-2) appears to be associated with PM, which can increase the severity of COVID-19 outcomes [128]. In particular, the spread of COVID-19 has been observed in northern Italian cities, where daily PM concentrations are higher than the annual average [129]. Like other viruses, SARS-CoV-2 could spread rapidly through the aerosol by utilizing the PM as a carrier. Notably, SARS-CoV-2 RNA was detected using three viral markers of PM in the area, thus suggesting a potent simultaneous exposure to SARS-CoV-2 and hazardous PM chemicals. Moreover, populations that live in areas with high levels of PM would be susceptible to acute or chronic inflammatory states owing to the dysregulated immune function induced by toxic chemicals released by PM. PM-insulted immune system would be inefficient in defending against viral infection [130]. In addition to the impaired antiviral responses, PM-induced production of free radicals can contribute to lung cell toxicity during SARS-CoV-2 infection [131,132]. Moreover, PM-activated inflammatory cells and cytokines can exacerbate pulmonary inflammation and tissue injuries in patients with COVID-19 [131]. Long-term and short-term exposures to high levels of pollutants correlated with an increase in COVID-19 incidence worldwide [131]. However, additional issues need to be addressed, including the particle size to which the virus binds, and more importantly, the duration for which the virus remains active in different age groups, as elderly subjects were found to be increasingly susceptible to COVID-19 worldwide.

## 6. PM-Linked Hypersensitivity

Exposure to air pollution increases the risk of asthma, allergic rhinitis, atopic dermatitis (AD), and allergen sensitization. The effects of air pollution exposure can be displayed differently depending on the timing of exposure, individual sensitivities, and simultaneous exposure to allergens.

### 6.1. Effect on Acute and Chronic Hypersensitivity Diseases

Previous studies have suggested that PM can increase hyperresponsiveness in the respiratory system. Diseases representing respiratory allergies include allergic rhinitis and asthma. For instance, urban levels of air pollution can induce pulmonary hyperresponsiveness in rats; however, the effect was reversed under the nonpolluted condition [133]. Moreover, PM can enhance immunological responses and inflammatory reactions in the nasal mucosa, but not the development of allergic rhinitis. Short-term exposure to PM (PM_10_ and PM_2.5_) increased the exacerbation of asthma, as well as the number of emergency cases [134]. Although there is a lack of evidence illustrating that PM can cause respiratory allergic diseases, such as asthma or allergic rhinitis, it is well accepted that PM is associated with exacerbations of asthma or allergic rhinitis [134]. It needs to be clarified whether short-term, peak exposure, or long-term exposure to PM is greatly associated with asthma exacerbation. Furthermore, PM exposure can induce allergic inflammation with T helper cell 2 (Th2) and Th17 differentiation, which can exacerbate allergic asthma [135]. Additionally, it can increase the risk of hospitalization in COPD. COPD is not a disease that is induced by hyperresponsivity—but following hyperactivity in the airways of COPD patients, disease exacerbation can occur because hyperactivity induces airway inflammation, thus narrowing the airway. Hence, COPD can be exacerbated by PM-associated hypersensitivity.

### 6.2. PM Enhances Allergen Sensitization

PM can enhance the immune response to specific allergens and increase IgE production. In a previous study, exposure to DEP increased IgE production in the nasal mucosa [136]. DEP exposure can only induce the production of IgE, but not of IgA, IgM, IgG, and albumin. In the lavage fluid, IgE-secreting cells were reportedly increased, but the number of IgA-secreting cells was not elevated. Additionally, epsilon mRNA production was altered. These results suggest that the local production of IgE is altered based on both quantitative and qualitative aspects, confirming that DEP can be an allergen and induces allergen specific IgE production. Moreover, ragweed specific IgE production was more highly induced upon a challenge of DEP and ragweed allergens when compared to that with ragweed allergens alone [137]. DEP and aeroallergens demonstrated synergistic effects and promoted allergen-induced allergic respiratory disease. Therefore, it can enhance allergen specific IgE production. Notably, DEP can elicit symptoms, act on mast cells, and enhance allergic responses, presenting more severe symptoms following high-dose, short-term exposure. These findings demonstrate that DEP can further augment allergic responses to other allergens.

Air pollutants, such as PM, can enhance allergen sensitization by carrying aeroallergens into the airway. Following the introduction of aeroallergens into the airway, airway epithelial cells promote oxidative stress and inflammation, increasing the antigenicity of allergens [138,139,140,141]. DEP alone did not induce the release of histamine and symptoms; the DEP and allergen + placebo groups demonstrated extremely low histamine levels. However, the histamine level increased by approximately three-fold in the DEP and allergen groups when compared to that in the allergen alone group. This implies that DEP did not induce an allergic response alone, but can increase the allergic response to the allergen, increasing the severity. Therefore, according to this study, PM does not act as an allergen alone, but augments allergic responses to other allergens, and hence, is harmful and can exacerbate other allergic diseases.

### 6.3. Effect of Prenatal and Early-Life Exposure on Hypersensitivity Diseases

It is well known that early-life exposure to fine PM in the air is associated with adult infant respiratory diseases, such as pneumonia and childhood asthma, with additional evidence suggesting that UFPs may affect childhood respiratory disease. PM can exacerbate respiratory allergic diseases, such as asthma and allergic rhinitis. In clinical evaluations, exposure to PM_10_ in the first year of life increased the incidence of asthma at 12 years of age [142]. Moreover, subjects born at locations with high PM_2.5_ exposure tend to display an increased incidence of asthma at seven years of age [143]. However, extensive investigations are warranted, as several studies have observed no correlation between the prevalence of allergic rhinitis or asthma incidence and PM exposure [144,145].

Several epidemiological studies have reported that living in places with high levels of air pollutants from birth increases the risk of allergic disease [91,108,144,146]. Additionally, if the mother resided adjacent to the main road, the offspring presented an increased risk of allergen sensitization and asthma. Studies have shown that exposure to air pollution during growth, even at an extremely young age, can affect lung function in adults. An 8-year follow-up of 1,759 students aged between 10 and 18 years in 12 schools in California, USA, revealed that those highly exposed to air pollution showed poorer lung functions than those who were not highly exposed, with 4.9 times higher risk [147]. Furthermore, a large cross-sectional study investigating children aged 6–14 years, in 10 cities in South Korea, observed that exposure to car-related air pollution increased the risk of asthma, rhinitis, allergen sensitization, and lowered lung functions in children living adjacent to major roads. In addition to subjective symptoms, effects on objective indicators of allergic diseases were recorded [146]. Moreover, short-term exposure to ambient temperature, relative humidity, diurnal temperature range (DTR), rainfall, and air pollutants, such as PM, were significantly associated with AD symptoms in infants and young children living in a temperate region [148]. The symptoms, including itching, sleep disturbance, erythema, dry skin, oozing, and edema, were significantly higher in subjects exposed to PM_10_ than in the control.

Asthma is characterized by variable airway obstruction, hyperresponsivity, and airway inflammation. In addition, asthma is a representative disease associated with hyperresponsiveness. However, the contribution of PM to asthma development remains inconsistent. Many birth cohort studies have been conducted. For example, in a cohort study in Asia, children with increased exposure to PM during gestational weeks 6–22 or the postnatal period demonstrated a higher incidence of asthma [149]. In this study, PM_2.5_ exposure in both the prenatal and postnatal periods induced later development of asthma. However, another study suggests that there is no significant correlation between the incidence of asthma or wheezing and exposure to PM_10_ and nitrogen dioxide (NO_2_) [150], with the prevalence of asthma determined as 15.2–23.3%. The prevalence was the lowest at age three and the highest at age five. The mean exposure to NO_2_ decreased from the first year of life to the eleventh year of life; the mean exposure to PM_10_ reportedly decreased.

### 6.4. Molecular Etiologies of PM-Linked Hypersensitivity

#### 6.4.1. PM-Derived Oxidative Stress

**Figure 3:**
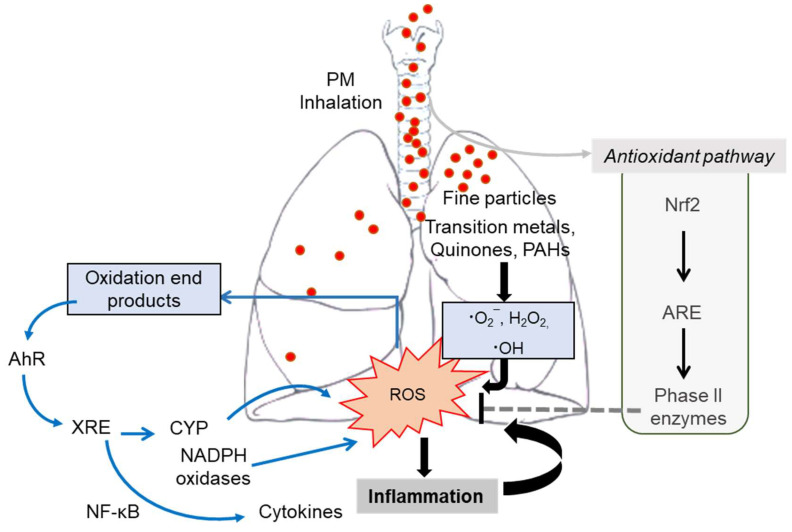
Schematic diagram of mechanisms of PM-induced oxidative stress and inflammation in the lung. When PM is inhaled, oxidative stress is initially triggered in the airways. Redox-active chemicals are transition metals, quinones, and organic components like PAH, which are responsible for the generation of ROS and stimulate the production of endogenous through AhR-induced CYP and NADPH oxidase. Moreover, the AhR-XRE system can enhance the production of proinflammatory cytokines. In spite of the Nrf2-ARE-associated antioxidant system for antioxidant enzymes and phase II enzymes, it fails to activate protection against the oxidative and proinflammatory burst, leading to lung and extrapulmonary tissue damages during PM-induced hypersensitivity.

PM causes oxidative damages in the pulmonary system [151]. The main adverse outcomes from exposure to PM are associated with oxidative stress from the disruption of oxidant redox-cycling reaction in the biological system. Although the pulmonary tissue contains the antioxidant defense systems [152], the generation of reactive oxygen species (ROS) plays pivotal roles in the pathologic process during PM exposure. PM can pose ROS in itself [50], or endogenous ROS can be generated through catalytic activity in vivo via cellular redox reactions stimulated by redox-active components of PM [151]. ROS are highly reactive molecules, including superoxide radical (O_2_^¯^), hydrogen peroxide (H_2_O_2_), and hydroxyl radical ∙OH [50]. Among ROS radicals, ∙OH is one of the most dangerous molecules that can react quickly with DNA and induce damage [153]. PM can catalyze ·OH generation mainly from transition metals and quinones [151], which are expected to affect the upper airways (75%) and lower parts in the lung (42–46%), respectively. PM generates hydroxyl radicals mostly sourced from biomass burning, incomplete combustion, and mineral dust [151,154,155]. Moreover, PM-derived redox-active components and oxidation products of the lipid membrane can serve as ligands for the aryl hydrocarbon receptor (AhR) via modulation of the xenobiotic responsive element (XRE) [135]. AhR-XRE signaling mediates the expression of ROS-producing metabolic enzyme systems, including cytochrome p450 monooxygenase (CYP), proinflammatory cytokines, and NADPH oxidase in the airways [156,157,158,159]. CYP and NADPH oxidase are pivotal players in endogenous production of ROS during PM exposure (Figure 3). Mechanistically, in addition to DNA damage and cytotoxicity, ROS and inflammatory insult can cause mitochondrial injury, which is the crucial step of pulmonary and cardiovascular distress during exposure to air pollutants [113,160].

Following exposure to air pollution, such as fine dust, the immune response to oxidative damage is altered in airway epithelial cells, with inhibited phagocytic action inducing various inflammatory reactions to increase inflammatory mediators, and reduced inflammatory damage in the airway [12,13,33]. In response to PM-induced oxidative stress, antioxidant defense systems are also triggered by NF-E2-related factor 2 (Nrf2), a master transcription factor via modulation of the antioxidant responsive element (ARE) [161]. The Nrf2-ARE-associated antioxidant system fails to activate protection against the oxidative and proinflammatory burst, leading to lung and extrapulmonary tissue damages during PM-induced hypersensitivity (Figure 3). In a cohort study, air pollution levels and lung function were evaluated in children in areas presenting severe air pollution in South Africa, revealing that decreased lung function was associated with mutations in the glutathione S-transferase P gene (*GSTP1*), which is involved in the metabolism of the antioxidant glutathione following air pollutant exposure [162]. When the gene is mutated, the effective antioxidant reaction against oxidative damage induced by air pollution is downregulated. ROS induces direct damage to proteins and lipids, impairs skin barrier function, and exacerbates atopic dermatitis (AD). In animal studies, AD exacerbation, resulting from oxidative skin damage, has been reported following air pollution exposure and the Th2 immune response [148].

#### 6.4.2. Inflammatory Insults

Increased expression of inflammatory mediators in the airway following exposure to air pollution causes an imbalance in immune responses and increases the likelihood of developing allergic diseases [113,163,164]. In 2003, Lambert et al. showed that exposure to fine dust activated the Th2 immune response after RSV infection, instead of before the usual viral defense mechanisms [165]. The concentrations of inflammatory cells in the bronchoalveolar fluid and Th2 cytokines, such as IL-13, were compared between RSV infected mice exposed to fine dust and RSV infected mice without fine dust exposure. Notably, a lower Th1 immune response was observed in animals presenting RSV infection and fine dust exposure. Exposure to fine dust resulted in an imbalance in the Th1/Th2 immune response after RSV infection, increased airway inflammation, and presented characteristic phenomena of asthma, such as airway sensitivity. It was confirmed that allergen specific Th2/Th17 cells increased in the lung and induced allergen reminiscence [166]. In the Cincinnati Childhood Allergy and Air Pollution Study, children were reportedly sensitized to inhaled allergens. When exposed to high levels of air pollution early in life, sensitization is more pronounced, and the prevalence of asthma is increased. However, there is no evidence supporting clinical outcomes in children. In particular, simultaneous exposure to allergens and air pollution during the early postnatal period amplifies allergic recall and promotes the development of asthma.

In addition to PM, various other matters are known to promote and exacerbate asthma; however, the pathogenesis of asthma induced by these compounds remains unclear. Airway macrophages could react quickly to the inhaled PM as an initial barrier. In patients with severe asthma, airway macrophages are unable to perform their functions, including the phagocytosis of inhaled PM. Th2 cells are essential for IgE isotype switching in B lymphocytes, which may stimulate allergic inflammatory responses, as well as airway hyperresponsiveness [167,168]. Furthermore, PM exposure increases the level of proinflammatory cytokines and induces macrophage polarization, especially inflammatory M1 polarization through the ROS pathway [169]. In certain situations, PM can induce M2 polarization. PM exposure has been associated with chronic inflammatory and autoimmune diseases. Typically, macrophages are cells related to the modulation of chronic inflammation, along with various infectious and autoimmune diseases. Depending on the type of stimulus, macrophages can be polarized into distinct functional phenotypes: M1-type proinflammatory/microbicidal cells, M2-type anti-inflammatory/suppressor cells, or a mixed state of activation [170]. Reportedly, owing to innate immunity, PM can be related to several diseases, including asthma and tuberculosis. Nevertheless, information regarding the pathophysiological aspects of these diseases and their association with innate immunity remains scarce. Various factors could influence the incidence, severity, and pathogenesis of these diseases, including the period of exposure, exposure duration, particle size and components of PM, ethnicity, age, and gender [36].

#### 6.4.3. Crosstalk with Allergens

Air pollution increases the permeability of airway epithelial cells and increases sensitization by increasing the exposure of immune cells or antigenicity of external allergens [171,172]. Although allergic rhinitis and AD have not been extensively associated with PM exposure, the inflammatory responses through the nose and skin can be expected to promote sensitization. Air pollutants play an important auxiliary role in allergen sensitization, including DEP and PAH, which are components of fine dust and automobile exhaust. DEP, a major component of vehicle exhaust, increased the allergenicity, inducing allergen sensitization through increased Th2 cytokines and mast cell activation [166]. In the US, a 5-year follow-up was performed in children between the ages of 0–5 years living adjacent to the main road. Reportedly, the group exposed to air pollution demonstrated higher serum total IgE levels than those not exposed to air pollution. It can be interpreted that during the process of allergic development, allergens with air pollution-related substances are carried into the airway and cause sensitization. Another study has reported that the concentration of Th2 cytokines in rhinorrhea increased by more than 16-fold following simultaneous exposure to pollen and DEP when compared with exposure to pollen allergens alone [166], suggesting PM-induced crosstalk with allergen responses.

#### 6.4.4. Epigenetics and miRNA Regulation during PM-Linked Hypersensitivity

Environmental exposure during pregnancy or early childbirth is closely linked to the development of allergic diseases, and in recent years, the mechanism has been gradually revealed in the development of welfare genetics. Welfare genetic changes are those in which the gene itself does not change owing to environmental exposure, but changes are observed in gene expression and activity. Changes in gene expression due to environmental exposure can occur over life, but it is postulated that effects are further pronounced in babies and infants. Studies have investigated fetal exposure to harmful environments, reporting that changes in the asthma risk may be substantiated by, for example, exposure to car exhaust and passive smoking in the womb, along with the risk of developing allergic diseases [173]. Exposure to a harmful environment during pregnancy acts on regulatory T cells and induces DNA methylation of allergy-related substances, such as FoxP3, contributing to the development of allergic diseases [174].

In genetically predisposed individuals, allergic diseases are susceptible to interaction with environmental factors. In particular, the inflammatory regulatory response in allergic diseases is presumed to involve gene regulation of the relevant immune system component; however, it has been recently noted that miRNAs affect the expression of these genes. miRNAs are extremely small noncoding RNAs, only 21–25 bases in size, that regulate gene expression by binding to mainly specific mRNAs, which inhibit or promote protein synthesis of target genes [175]. Reportedly, miRNAs regulate several genes in cells, thereby regulating immune cell growth, differentiation, and proliferation in allergic diseases. To date, several miRNAs associated with AD have been revealed, with dysregulation, such as increased or decreased miRNA expression in patients with AD when compared with healthy, atopic skin. Furthermore, studies have shown that the number of low regulatory T cells in cord blood was associated with increased miRNA-223 expression, affecting atopic pathogenesis [176].

## 7. Conclusions

In this review, we systemically discussed the immune alteration-associated disease outcomes with cellular and molecular evidence in response to PM, including biomass-derived pollutants. Epidemiological and experimental studies have demonstrated that all particles are not equally toxic, but cause different adverse health effects. Generally, the particulate size is the deterministic factor of translocation and actions in the airway mucosa and other tissues. Although most fine particles deposits in the air-blood barrier tissues, UFPs, including some components of WSP, can easily migrate to different regions of the body, leading to local and systemic immunological responses. Although the migration of most fine particulates to the circulation is limited, PM-derived or endogenously produced ROS and inflammatory cytokines can mediate acute and chronic distress, including infection and hypersensitivity during PM exposure.

Despite controversies about whether PM can either stimulate or inhibit adaptive immune responses, PM-induced cellular, and molecular alterations can contribute to outcomes of human diseases, including infection, hypersensitivity, and chronic diseases depending on chemical composition or size of the PM and exposure regimes (frequency, duration, and age). Exposure to PM, including WSP, alters the macrophage activity through the pattern-recognition signaling, leading to the production of proinflammatory cytokines and activation of innate immune cells in the airway and circulation. Depending on the exposure regimes, differentially activated macrophages play crucial roles in disease development and progression via specific molecular pathways. In particular, exposure to PM_2.5_ induces M1 macrophage-dependent inflammation by increasing the secretions of Th1 and Th17 cytokines through PRR-activated signaling, while chronic exposure to PM_2.5_ can reprogram cellular polarization toward M2 macrophage-involved sustained inflammation, fibrosis, and deleterious airway remodeling. In terms of host responses to infection, PRR-activated macrophages produce M1-like proinflammatory cytokines, destroying the invading pathogens. However, some bacterial pathogens tend to prevent M1-like polarization, facilitating M2-like transition during the chronic inflammation and fibrosis in association with polarization toward Th2-lined immune response to PM exposure.

However, there are still a few questions that need to be addressed for a more comprehensive understanding. First, the mechanistic implications of the immune response and mode of action of different PM mixtures have not been fully elucidated with comparative investigations for diverse exposure regimes. In addition to individual chemical-specific toxicity, integrative effects of PM mixture on human immunity-related disease processes need to be further investigated. In particular, clinical evaluation of simultaneous exposure to the emerging microbes and air pollutants are increasing in interest. Epidemic investigation in the population under the biomass-associated air pollution would provide crucial insights to predict unexpected risks in an emerging microbe-abundant society. Second, target cellular molecules linked with immune dysregulation mediated by PM actions remain elusive. The identification of target molecules and their mechanistic network would help identify new strategies to inhibit the adversary health effects of biomass-derived PM, including WSP. The present review provides insights into novel immunotherapeutic interventions against PM-induced acute and chronic environmental disorders. Moreover, it is warranted to address climate change- or deforesting-associated PM exposure and health for the sustainable planet.

## Figures and Tables

**Table 1:** Main characteristics, primary sources, and properties of each particulate matter (PM) fraction.

Sl. No.	Characteristics	Ultrafine Particles (PM_0.1_)	Fine Particles (PM_2.5_)	Coarse Mode Particles (PM_10_)
1.	Diameter	≤ 0.1 μm	0.1–2.5 μm	2.5–10 μm
2.	Sources	Diesel and automobile exhaust, emissions from the combustion of gas stove, vented gas dryer and candle, electric motors, residential burning	Emissions from the combustion of gasoline, oil, diesel fuel, wood burning, and coal burning	Abraded soil, dust from road and construction sites, landfills and agriculture, wildfires and brush/waste burning, industrial sources, fungi and bacteria, endotoxins, and pollen
3.	Atmospheric half-life	Minutes to hours	Days to weeks	Minutes to days
4.	Ability to travel (km)	1 to 10	100 to 1000	1 to 100
5.	Redox activity	High	Medium	Low
6.	Transition metal	Low	High	Medium
7.	Polyaromatic hydrocarbon	High	Low	Low
